# Identification of the subtypes of gastric cancer based on DNA methylation and the prediction of prognosis

**DOI:** 10.1186/s13148-020-00940-3

**Published:** 2020-10-28

**Authors:** Tengda Li, Xin Chen, Mingli Gu, Anmei Deng, Cheng Qian

**Affiliations:** 1 State Key Laboratory of Experimental Hematology, National Clinical Research Center for Blood Diseases, Institute of Hematology & Blood Diseases Hospital, Chinese Academy of Medical Sciences & Peking Union Medical College, Tianjin, 300020 China; 2 Princeton High School, 151 Moore Street, Princeton, NJ 08540 USA; 3 Department of Laboratory Diagnosis, Changhai Hospital, Navy Military Medical University, Shanghai, 200433 China; 4 Changhai Hospital, Navy Military Medical University, Shanghai, 200433 China; 5 Department of Laboratory Medicine, Shanghai Municipal Hospital of Traditional Chinese Medicine, Shanghai University of Traditional Chinese Medicine, Shanghai, 200071 China

**Keywords:** Gastric cancer, DNA methylation sites, Prognosis, Diagnosis, TGFβ2

## Abstract

**Background:**

Gastric cancer (GC) is a digestive system cancer with a high mortality rate globally. Previous experiences and studies have provided clinicians with ample evidence to diagnose and treat patients with reasonable therapeutic options. However, there remains a need for sensitive biomarkers that can provide clues for early diagnosis and prognosis assessment.

**Results:**

We found 610 independent prognosis-related 5′-cytosine-phosphate-guanine-3′ (CpG) sites (*P* < 0.05) among 21,121 sites in the training samples. We divided the GC samples into seven clusters based on the selected 610 sites. Cluster 6 had relatively higher methylation levels and high survival rates than the other six clusters. A prognostic risk model was constructed using the significantly altered CpG sites in cluster 6 (*P* < 0.05). This model could distinguish high-risk GC patients from low-risk groups efficiently with the area under the receiver operating characteristic curve of 0.92. Risk assessment showed that the high-risk patients had poorer prognosis than the low-risk patients. The methylation levels of the selected sites in the established model decreased as the risk scores increased. This model had been validated in testing group and its effectiveness was confirmed. Corresponding genes of the independent prognosis-associated CpGs were identified, they were enriched in several pathways such as pathways in cancer and gastric cancer. Among all of the genes, the transcript level of transforming growth factor β2 (TGFβ2) was changed in different tumor stages, T categories, grades, and patients’ survival states, and up-regulated in patients with GC compared with the normal. It was included in the pathways as pathways in cancer, hepatocellular carcinoma or gastric cancer. The methylation site located on the promoter of TGFβ2 was cg11976166.

**Conclusions:**

This is the first study to separate GC into different molecular subtypes based on the CpG sites using a large number of samples. We constructed an effective prognosis risk model that can identify high-risk GC patients. The key CpGs sites or their corresponding genes such as TGFβ2 identified in this research can provide new clues that will enable gastroenterologists to make diagnosis or personalized prognosis assessments and better understand this disease.

## Background

Gastric cancer (GC) is one of the leading causes of cancer-related deaths worldwide. Patients are often diagnosed with GC at an inoperable stage, and recurrence is common after resection [1]. The mortality rate for GC ranks second among all cancers globally [1]. Traditional classification methods for GC are primarily based on the anatomic or histological features [2, 3]. The main cause of GC is helicobacter pylori (H. pylori) infection, which may lead to chronic inflammation and consequently tumorigenesis [4]. In addition to H. pylori infection, other risk factors such as environmental, genetic, and epigenetic factors have also been identified [5]. Although the etiology, clinical characteristics, and classifications have previously been described in detail, the same treatment methods are routinely applied for all patients with GC despite the complex genetic heterogeneity of the disease, which can result in poor prognosis. Therefore, specific biomarkers that can help clinicians better understand the mechanism of GC and develop personalized treatments are needed.

Epigenetic changes mainly include DNA methylation, non-coding RNA, chromatin remodeling, and histone modifications [5]. These factors can regulate the expression of genes without DNA sequence changes and are inheritable across generations [5]. DNA methylation is a well-characterized epigenetic modification and plays an important role in carcinogenesis [6, 7]. It is mediated by DNA methyl-transferase (DNMT), which transfers methylated groups from S-adenosyl-*L*-methionine (SAM) to the pyrimidine ring of cytosine residues on DNA [8]. The methylation reaction often occurs on the cytosine of 5′-cytosine-phosphate-guanine-3′ (CpG) dinucleotide, with few methylations on non-CpG sequences [9]. Previous studies showed that DNA hypermethylation at promoter regions could silence the expression of the targeted genes, further having influence on the bioprocesses such as cell cycles, DNA repair, and even the signaling pathway for tumor development, indicating that it might play an important role in promoting cancers [7, 10–12]. Abnormal changes of DNA methylation or demethylation exist in different tumor suppressor genes or oncogenes [5]. These changes often occur before tumor formation or development and can be considered as early diagnostic tumor markers or the predictors for people with high risk of developing cancer [5, 7, 13, 14]..

Studies on nasopharyngeal carcinoma have demonstrated that methylation in promoters of the Ras association domain family 1A and disheveled-associated binding antagonist of β-catenin 2 can help clinicians perform early diagnosis [15, 16]. Teng et al. [17] reported that inter- and intratumor DNA methylation heterogeneity in esophageal squamous cell carcinoma was associated with lymph node metastasis and patient prognosis. For GC, although several CpGs sites had been found involving in the processes of GC development including invasion and metastasis [5], there was no systematical analysis to establish an efficient model which could identify patients with high risk of poor prognosis. Here, we analyzed the methylation data from more than 400 GC samples, in order to find the key prognosis-related methylation biomarkers and establish a prognosis assessment model, providing more evidences for doctors to perform early diagnosis and prognosis assessments.

## Methods

### Data download and preprocessing

Three hundred and forty-three transcript files for primary GC were obtained from the cancer genome atlas (TCGA) repository (https://portal.gdc.cancer.gov/, 2020-04-01), and the platform of gene expression RNAseq was Illumina. The information for 406 patients and their follow-up data are downloaded (https://portal.gdc.cancer.gov/, 2020-04-02) and presented in Additional file 1: Table S1. Methylation data were obtained from the University of California Santa Cruz (UCSC) Cancer browser (https://xena.ucsc.edu/, 2020-04-01). In total, 470 DNA methylation files were downloaded, among all of the methylation files 397 DNA methylation files were from Illumina Human Methylation 450 platform and 73 files were from Illumina Human Methylation 27 platform, the overlapped methylation sites of the two platforms were selected for further study. Because one patient might have two DNA methylation files, the number of DNA methylation files was more than the number of the patients, while because some patients might not have transcript files, the number of transcript files was less than the number of GC patients.

The criteria for deletion of the methylation sites were as follows: The site had missing values in more than 70% of the samples; it was located in the sex chromosomes; single-nucleotide polymorphisms; CpGs above 2 kb upstream to 0.5 kb downstream (gene promoter regions); and cross-reactive genome CpG sites [18]. The clinical samples would be excluded if: its follow-up duration was less than 30 days, or there was no follow-up data; it was lack of survival status; its clinical data such as TNM staging system (T indicates the extent of the primary tumor, N indicates lymph node involvement, and M indicates distant metastases), grade, age, tumor stage, and gender were missing or unknown. The Impute R package and ComBat algorithm in the sva R package were used for batch corrections [19–21]. We divided them randomly into two groups (training group and testing group). We selected the intersection of the training dataset (Additional file 2: Table S2) and the testing dataset (Additional file 3: Table S3) to perform the further study; thus, the model created with the training dataset could be applied to the testing.

### Determination of classification features for methylation sites by Cox proportional risk regression analysis

The methylation levels for the CpG sites, age, stage, gender, tumor classifications (T, M, N), grade, and follow-up data were used to create univariate Cox proportional risk regression models from which 1054 significant CpG sites (*P* < 0.05) were obtained (Additional file 4: Table S4). These significant sites were imported into multivariate Cox proportional risk regression models. We selected the CpG sites that were significant in both models as the characteristic methylation sites (*P* < 0.05, Additional file 5: Table S5) that could significantly affect the survival of patients with GC.

### Determination of prognosis-related methylation subtypes

The CpG sites shown in Additional file 5: Table S5 were analyzed using the ConsensusClusterPlus package [22] in R in order to define the GC subtypes. Each sample in the data we collated was divided into k groups based on the mean k values (k-means), and the number of times this process was repeated was used to establish consensus values and evaluate the stability of the classification sets. The calculated pairwise consensus values were recorded in a consensus matrix for each k value. Pairwise consensus values were defined as the proportion of two subjects that appeared in the same cluster compared to the number of times they appeared in the same subsample. The final results from agglomerative hierarchical consensus clustering based on 1-Pearson correlation distances were also divided into k groups. Eighty percent of the data for the tumor samples were calculated in each iteration. The Euclidean squared distance metric was applied in the k-means calculation, and more than 100 iterations were included in the results matrix. The exported graphs contained the consensus matrices, consensus cumulative distribution function (CDF) plot, delta area plot, and tracking plot. We determined the k value if there was a low relative change in the area under the CDF curve, relatively high conformity in the clusters, and a low variation coefficient. The coefficient of variation was calculated as (standard deviation/mean number of samples) * 100%. The area under the CDF curve was described as the category number. The pheatmap package in R was used to create the heat map [23]. Squares were distributed diagonally in the map when the matrix consensus was perfect.

### Correlation between the molecular subtypes and survival or clinical features of GC patients

The Survival package [24–26] in R was used to perform survival analysis for GC patients whose subtypes were determined from the methylation profiles and the results are shown in Kaplan–Meier plots. The log-rank test was applied to compare the methylation levels in different clusters. The Chi-squared test was used to find the differences in categorical data as the clinical characteristics or other features among these subtypes. The tests were two-sided with a significance level of 0.05.

### Prognostic risk model construction and assessment

Based on the methylation levels of the CpG sites in the cluster with relatively high or low survival probability and the follow-up information, the Cox proportional hazard model was created with the coxph function in the Survival R package [27, 28]. The formula for this model was: Risk score = ID_1 _* Coefficient_1_ + ID_2 _* Coefficient_2_ + ID_3 _* Coefficient_3_…… + ID_n_*Coefficient_n_, all of the methylation sites’ IDs and their coefficients are shown in Additional file 6: Table S6.

### Functional enrichment analysis

**Fig. 1:**
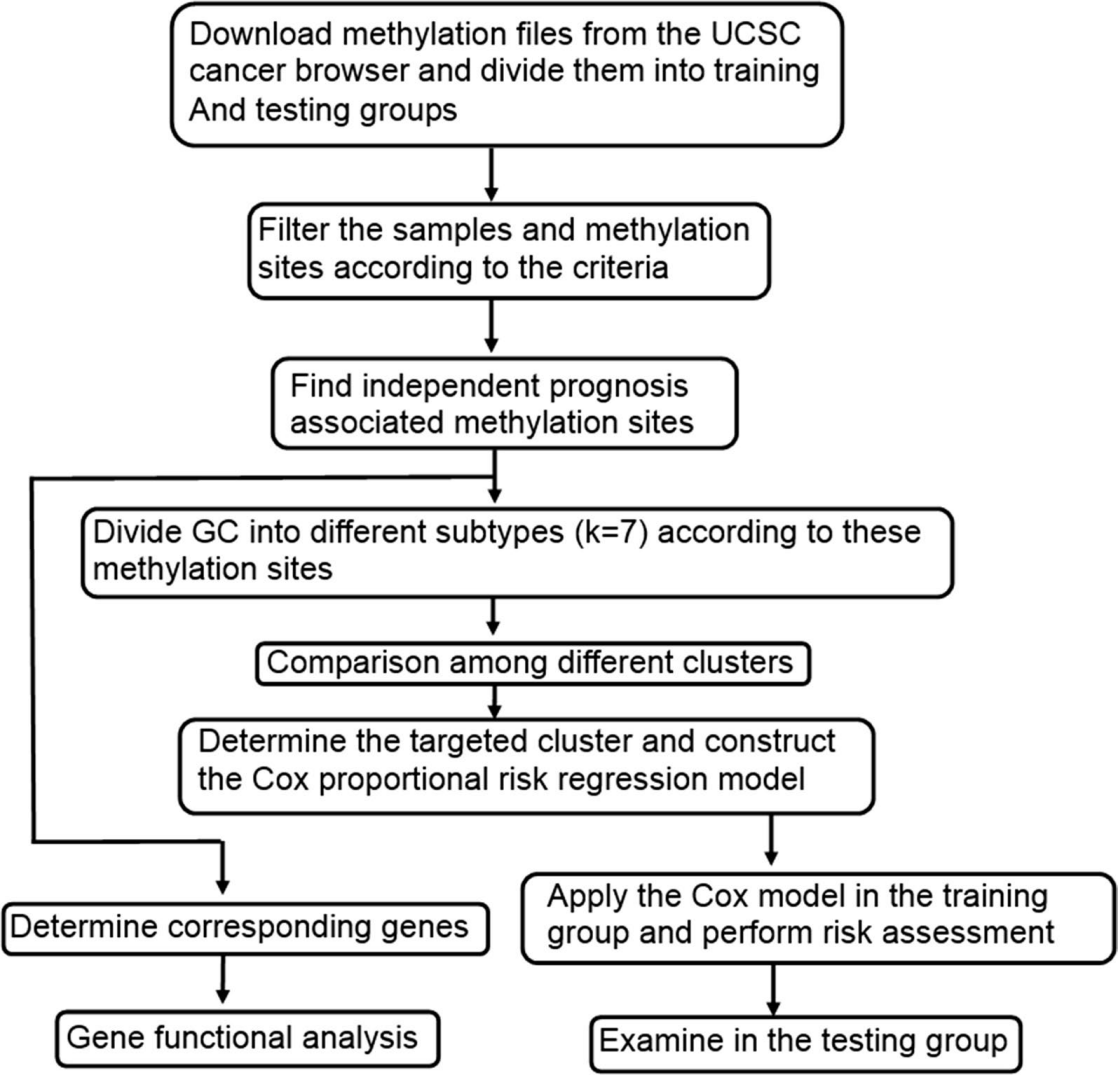
Diagram of study flow. UCSC, University of California Santa Cruz; GC, gastric cancer

We identified the gene symbols related to the methylated sites using the Strawberry Perl 5.30 software, and the symbols were transformed into gene ID. Kyoto Encyclopedia of Genes and Genomes (KEGG) analysis were performed by ClueGO [29, 30]. Tree interval was from 3 to 8, the minimal number of genes in pathways was 3. Network connectivity (kappa score) was 0.4, the statistical option was enrichment/depletion (two-sided hypergeometric test) and pV correction was Bonferroni step-down. The study workflow of this research is shown in Fig. 1.

### Statistics

The correlation between two continuous data was analyzed with Spearman’s correlation analysis if the data distribution was not normal, whereas Pearson’s correlation test was used for data with a normal distribution. Comparisons of two groups were made with the t-test or Mann–Whitney U test; if there were more than two groups, the comparison was made with analysis of variance (ANOVA) or Kruskal–Wallis test. The Wilcox test was used to compare the methylation levels between the seven clusters. All the statistical analysis results described below were obtained with IBM SPSS statistics 21.0 or the R software. The significance level was set to *P* < 0.05.

## Results

### Determination of prognosis-related methylation sites

The methylation data were downloaded and preprocessed as described in “Methods” section. Four hundred and six patients (150 women and 256 men) with GC were included in this study (Additional file 1: Table S1). Their mean age was 65.6 years. After conditional filtering and batch correction, the number of methylation sites in both the training and testing matrixes was 21,121 (Additional file 2: Table S2, Additional file 3: Table S3). The number of patients with grades 1, 2, and 3 tumors was 10, 149, and 240, respectively, while the number with stages I, II, III, and IV tumors was 56, 118, 167, and 39 (Additional file 1: Table S1). For the primary tumor status according to the TNM staging system, there were 23 patients with T1, 85 with T2, 185 with T3, and 103 with T4 (Additional file 1: Table S1). For the tumor metastasis status, the number of patients in the tumor M0 stage was 361, while 27 were in the M1 stage (Additional file 1: Table S1). For the lymph node involvement status, there were 122 patients with N0, 109 with N1, 80 with N2, and 78 with N3 (Additional file 1: Table S1). Using univariate Cox regression analysis, we found that 1054 methylation sites were related to patient prognosis (Additional file 4: Table S4). Multivariate Cox regression was used to analyze these methylation sites, and 610 independent prognosis-related methylation CpG sites were observed (Additional file 5: Table S5).

### Identifying clusters of GC by prognosis-associated DNA methylation sites and their relationships with clinical features

**Fig. 2:**
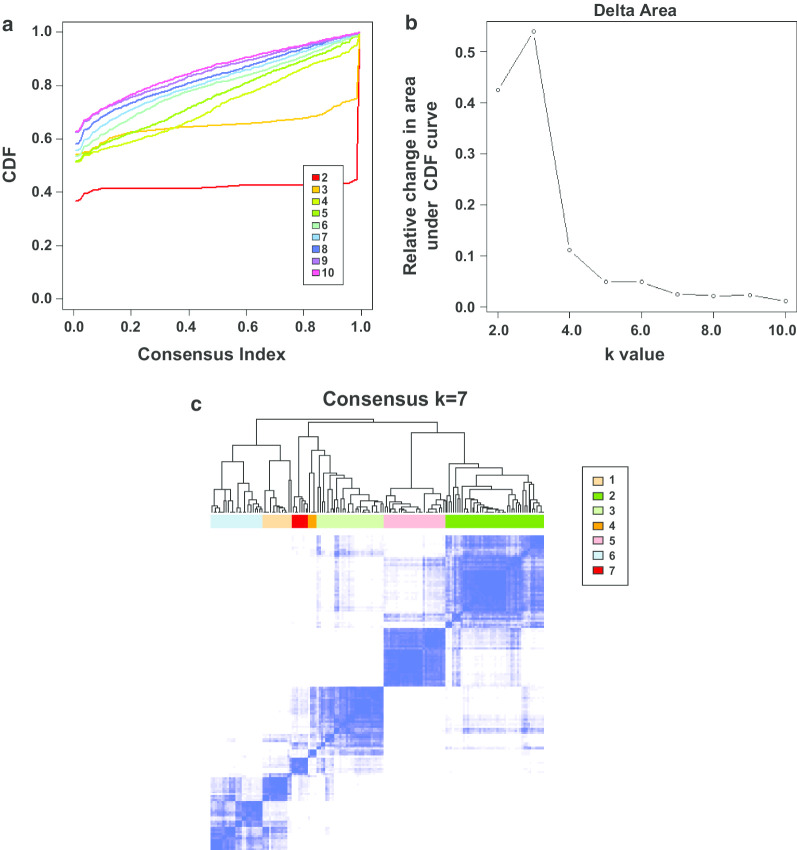
Consensus matrix of the clusters and the conditions for selecting the number of clusters. **a** Consensus Index-CDF graph. The number of clusters ranged from 2 to 10. **b** Delta area curves. **c** Consensus matrix when k = 7. Numbers 1 to 7 indicate the first to the seventh cluster. White is 0, and blue is 1. CDF, consensus cumulative distribution function; k, the number of clusters

**Fig. 3:**
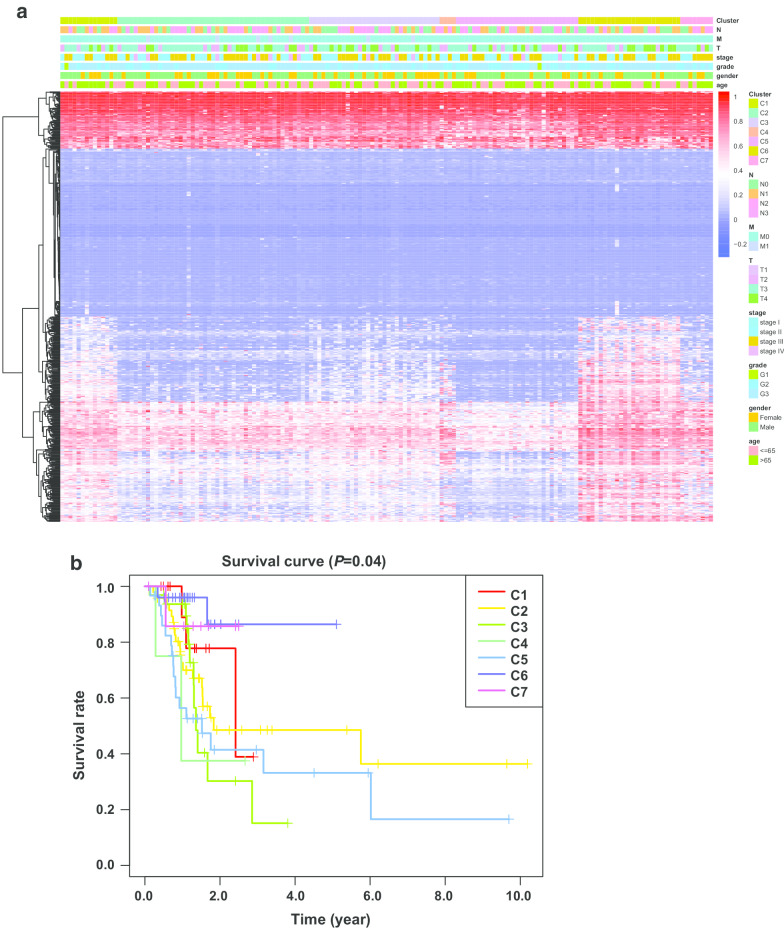
Heat map of methylated levels among different clusters and their clinical characteristics. **a** Heat map of the DNA methylation levels in the seven clusters and their clinical features. **b** Survival curves of different tumor subtypes. The unit of age is year. C, cluster; T, primary tumor; N, lymph node involvement; M, distant metastases; G, grade

After combining the CDF-consensus plot and delta area (Fig. 2a, b), we observed that when k = 7, there were relatively few changes in the area under the CDF curve and high conformity in the clusters with a low variation coefficient. The map of the seven clusters was mostly diagonal, indicating a good polymerization effect (Fig. 2c). Figure 3a shows that the methylation levels of the seven clusters were different and the methylation expression in cluster 6 was higher than that in the other clusters. The clinical features were evenly distributed in each cluster (Additional file 7: Figure S1, Fig. 3a). Survival curve analysis showed there was a significant difference between different categories (*P* = 0.04), which indicated that the classification system separated the patients into different subgroups with different prognoses (Fig. 3b). Cluster 6 had a higher survival rate than the other clusters (Fig. 3b). There was no difference in the seven clusters in terms of clinical features such as age (*P* = 0.593) and gender (*P* = 0.559). The detailed composition ratios for the clinical features in the seven clusters are shown in Additional file 7: Figure S1 and Fig. 3a, which indicate the differences in the prognosis for the seven clusters were not affected by clinical characteristics such as age and gender. In cluster 6, the clinical M status was M0, and the number of patients with N0 and T1 was greater in cluster 6 than in the other clusters (Additional file 7: Figure S1), indicating that patients in the other clusters had more severe disease than those in cluster 6.

### Comparison of levels of methylation in different clusters

**Fig. 4:**
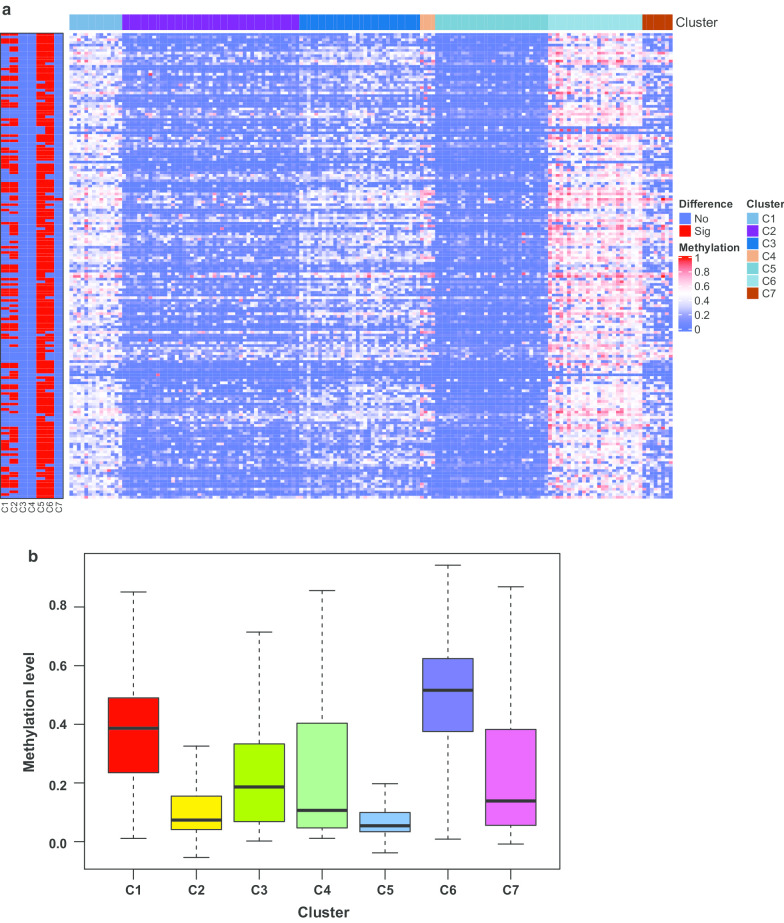
Levels of prognosis-related methylation sites of different gastric cancer subtypes. **a** Heat map of differentially expressed methylation sites in the seven clusters. The heat rectangle on the left map is red if there is a statistically significant difference between the selected cluster and the other clusters; otherwise, it is blue. Sig, significance, *P* < 0.05; No, no significance. **b** Comparison of the methylation levels between cluster 6 and other clusters. The methylation sites involved in this box plot were significantly altered in cluster 6 compared to the other clusters. C, cluster

The methylation levels of 610 independent prognosis-associated sites were compared among the seven tumor clusters in the training group (Additional file 8: Table S7). If there was statistical difference in the methylation levels between the selected cluster and the other clusters, the methylation site was chosen and the difference was presented in red (Fig. 4a). Otherwise, the methylation site was shown in blue (Fig. 4a). A total of 173 sites were altered in at least one cluster and these are shown in Fig. 4a. From the heat map, we observed the altered methylation sites were mainly located in cluster 6. The results of the Wilcox test showed that 149 methylation sites were altered in cluster 6 compared with the other clusters (fold change > 2, *P* < 0.05, Additional file 8: Table S7). Combined with the survival curve (Fig. 3b), which indicated that cluster 6 had a higher survival rate than the other clusters, we selected the different methylated expression files for cluster 6 to create a box plot (Fig. 4b). The methylation level was higher in cluster 6 than in any other cluster (Fig. 4b).

### Construction and efficiency of the prognosis risk model for GC

**Fig. 5:**
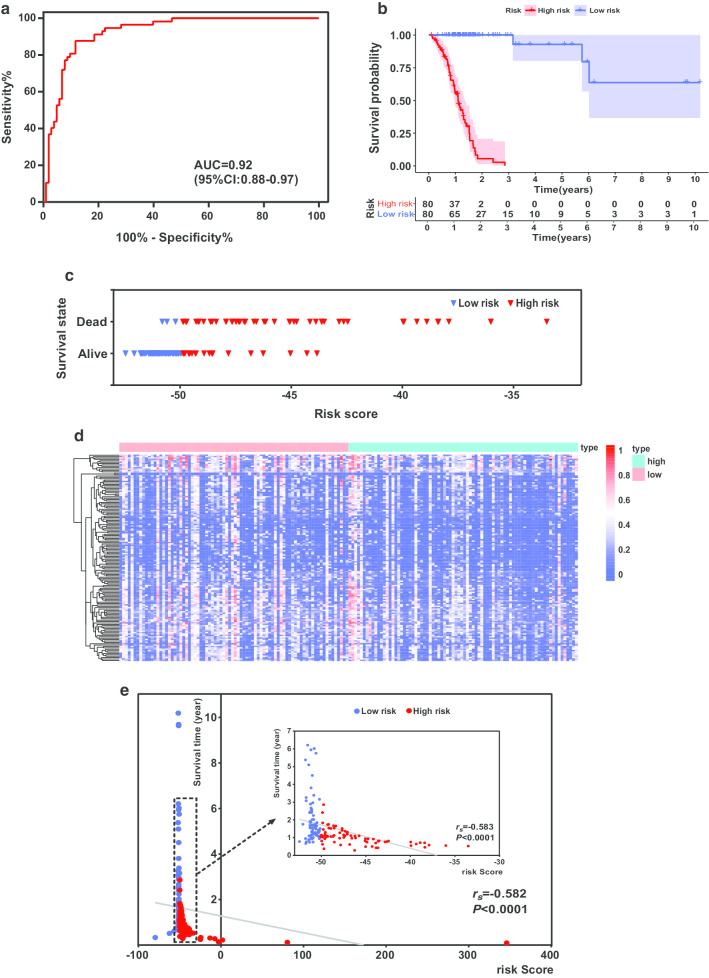
Risk assessment and efficiency of the established prognosis-predicting model in training group. **a** ROC curve for the results from constructed risk model. **b** Survival curves for high-risk and low-risk groups. **c** Survival state of low-risk and high-risk patients with increasing risk score. **d** Heat map for the levels of methylation sites used in the Cox risk regression model. **e** Survival time for every patient with increasing risk score. ROC, receiver operating characteristic; AUC, area under the curve; CI, confidence interval; *r*_*s*_, coefficient of Spearman correlation analysis

We assessed the efficiency of this approach for distinguishing high-risk cases from low-risk cases using receiver operating characteristic (ROC) analysis. The area under the ROC curve was 0.92 (95% confidence interval: 0.88–0.97, *P* < 0.0001), indicating that this model had good examining efficiency (Fig. 5a). The construction and computation processes for the prognosis risk model were performed as described in “Methods” section. We calculated the risk scores for all patients in the training group and ordered the patients according to their risk scores (Additional file 9: Table S8). The median risk score was − 50.07; patients with risk scores below the median value were classified into the low-risk group and those with higher scores were placed in the high-risk group (Additional file 9: Table S8). The survival curve showed that the high-risk group had a lower survival probability than the low-risk group overall (*P* < 0.0001, Fig. 5b). Figure 5c, d shows that as the risk score increased, the number of deaths increased, while the methylation levels of the selected sites decreased. Furthermore, we found a negative correlation between patients’ survival time and the risk score (Fig. 5e, *r*_*s*_ = − 0.582, *P* < 0.0001). After excluding the discrete data, the survival time of patients was also negatively correlated with the risk scores (Fig. 5e, *r*_*s*_ = -0.583, *P* < 0.0001). These results confirmed their negative relationship and indicated that patients with high risk scores had lower survival time than patients with low risk.

**Fig. 6:**
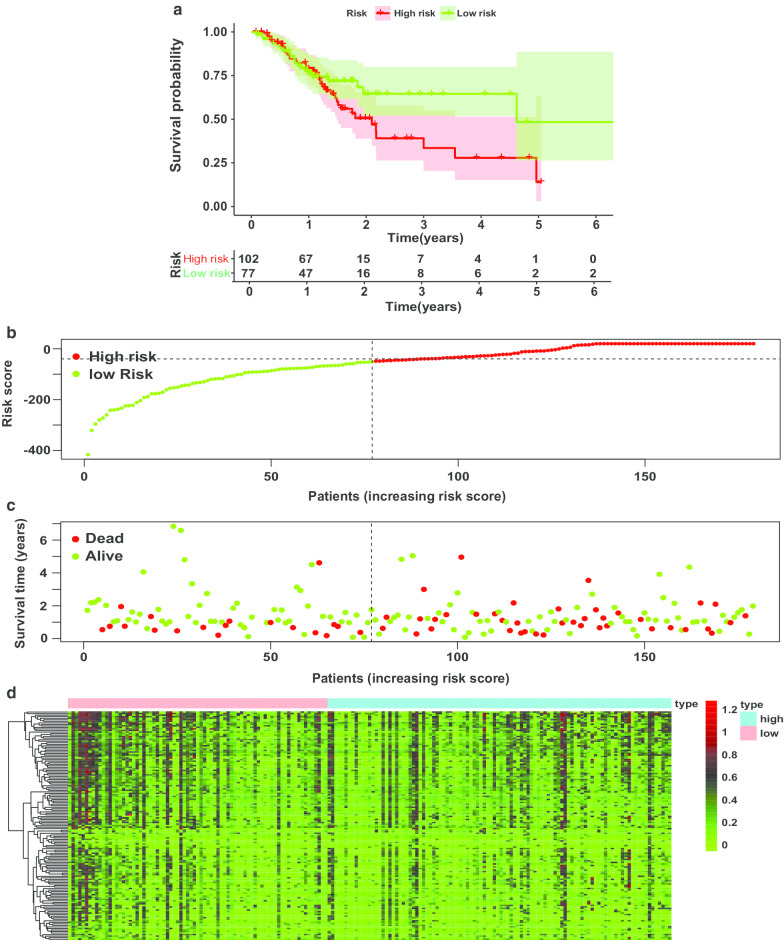
Validating the efficiency of the prognosis-predicting model in testing group. **a** Survival curves for high-risk and low-risk groups. **b**, **c** Prognosis risk plots. Risk score (**b**) and survival time**/**state (**c**) for every patient. **d** Heat map for the levels of methylation sites used in the Cox risk regression model

We next applied the constructed system in the testing group (Additional file 10: Table S9). From the survivorship curve, we could see that patients in high-risk group had lower survival probability than in low-risk group (Fig. 6a). With the increasing risk scores, the number of dead patients increased and the levels of the methylation sites in the Cox regression model decreased (Fig. 6b, d). The results demonstrated that the established model by training group could be validated in the testing group.

### Function enrichment analysis of the annotated genes for methylation sites

**Fig. 7:**
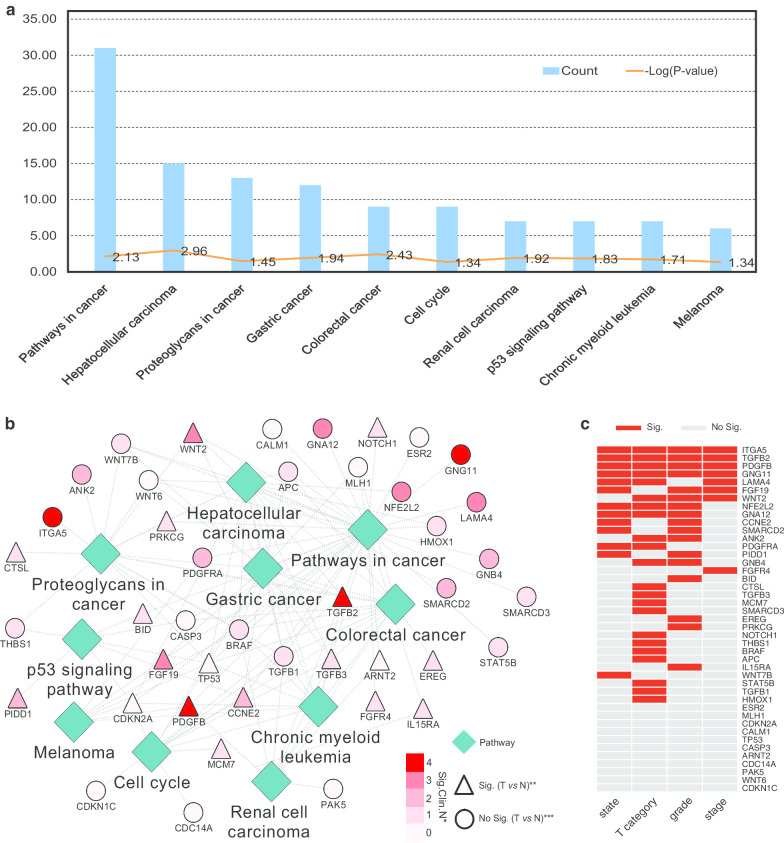
Functional enrichment analysis for genes corresponding to the independent prognosis-related methylation sites. **a** Enriched pathways of corresponding genes for the 610 independent prognosis-related methylation sites according to the Kyoto Encyclopedia of Genes and Genomes. **b** Network for the pathways.*The number of significant *P* values (< 0.05) for comparison of the gene expression levels among clinical categories of state, T, grade, and stage. For example, if the gene level is changed in different tumor states, T categories, grades, and stages, the number is 4, while if it changes just in different tumor stages, the number is 1; the detailed information is shown in **c**. **, the gene level is significantly different between patients with gastric cancer and the normal. ***, There is no significance between patients with gastric cancer and the normal. T, tumor; N, normal. **c** Results of comparison of the levels of site genes among different tumor states, T categories, grades, and stages. Sig., *P* < 0.05; No Sig., *P* > 0.05

Six hundred and seventy genes were found corresponding to the 610 independent prognosis-related methylation sites. Function enrichment analysis of these genes showed that they were mainly enriched in 10 pathways such as pathways in cancer, gastric cancer, and colorectal cancer, and all of the pathways and associated genes are presented in Additional file 11: Table S10 and Fig. 7a, b. The networks for the pathways and the involved genes are presented in Fig. 7b, from which we could see they were all related to the cancer processes and might involve in the pathological processes of GC; interestingly, we also found that TGFβ2 was included in 8 pathways, i.e., pathways in cancer, proteoglycans in cancer, hepatocellular carcinoma, gastric cancer, colorectal cancer, chronic myeloid leukemia, cell cycle, and renal cell carcinoma. We further compared the gene expression levels in Fig. 7b among different tumor stages, T categories, grades and patients’ states, and found that the levels of integrin subunit alpha 5 (ITGA5), TGFβ2, platelet-derived growth factor subunit B (PDGFB), and G protein subunit gamma 11 (GNG11) were changed with statistical difference in the above four terms (Fig. 7c, Additional file 12: Table S11). The expression of nuclear factor, erythroid 2-like 2 (NFE2L2), and G protein subunit alpha 12 (GNA12) were significantly different in clinical terms of survival state, T category, and grade, and detailed information for other genes is shown Additional file 12: Table S11. We also compared their transcript levels between the normal and the patients with GC, and found the gene expression levels such as TP53, TGFβ2 were significantly changed (*P* < 0.05), and detailed information is presented in Additional file 13: Table S12 and Fig. 7b.

## Discussion

GC accounts for 20% of the burden of disability-adjusted life-years from cancer worldwide [31]. It is a cancer with complex genetic heterogeneity and because of this, the classification of GC using Lauren’s or other histological criteria is ignored when treating the subtypes [3, 5, 32, 33]. Although cancer-related biomarkers and targeted drugs such as trastuzumab have been found and are used in clinical practice, the tumors are often inoperable when patients are diagnosed and can recur after resection [1]. To better understand the mechanism for GC and provide early diagnosis biomarkers, epigenetic biomarkers especially DNA methylation sites are critical [5, 6]. Specimens for the evaluation of DNA methylation can be obtained from the body fluids of patients through noninvasive procedures, providing a promising method for patient diagnosis at a relatively early stage without pain.

In this study, we analyzed the methylation data for GC based on a large number of clinical samples (235 files in the training group and 235 in the testing group) using systematic analysis methods. We constructed a Cox risk regression model based on training group as described earlier. This model could effectively predict the patients’ prognosis. Its efficiency could be validated in the testing group. Although the TCGA research network identified two different CpG island methylator phenotypes (CIMP) of gastric cancer: Epstein-Barr virus-CIMP (EBV-CIMP) and Gastric-CIMP, it did not reveal the relationship between the prognosis and the two methylator phenotypes; furthermore, the TCGA network did not do a systematical analysis to find the key CpGs or establish an efficient model to identify patients with poor prognosis [34–36]. In the research by Nikolay et al. [37], they aimed to find methylation biomarkers to solve the problem of the misclassifications of gastroesophageal junction tumors. For the data preprocessing, they excluded the probes that were not part of the IIumina Human Methylation 27k array probes and used multiple survival screening methodology to find the targeted CpGs such as cg26117023, cg0402816 and cg21475255, which were very close to their downstream transcription start sites (TSSs) [37]. In our study, we aimed to find the methylation sites that could predict the prognosis of patients diagnosed with gastric cancer. To achieve it, we merged the patients’ prognosis information such as the survival time or survival state with the methylation files and specially examined CpGs in promoter regions (2 kb upstream to 0.5 kb downstream); then, we used the multivariate Cox proportional risk regression models to find the independent prognosis methylation sites and divided the gastric cancer into 7 subtypes; these identified independent prognosis methylation sites were different from the sites in the study of Nikolay’s team [37]. Furtherly we analyzed the corresponding genes of the independent prognosis-related CpGs by ClueGo and found most genes were enriched in pathways in cancer, hepatocellular carcinoma, and gastric cancer (Fig. 7a, b), while in the research of Nikolay et al. [37], the identified methylation signatures were associated with protein binding, gene expression, and cellular component organization cellular processes. In the study of Hu et al. [38], they found DNA methylation gene signatures consisting of five genes (SERPINA3, AP00357.4, GZMA, AC004702.2, and GREB1L) as prognosis predictors with AUC of 0.72. As we know, one methylation site might corresponding to two genes, and it is not a one-to-one relationship; thus, we directly used the methylated level of the CpGs to predict the prognosis risk of every patient; this is more accurate and the AUC of our study is 0.92; it is much higher than the AUC of 0.72 in the study of Hu et al. [38]. Furthermore, we only included the CpGs in the region of gene promoter as described above, and the exclusion criteria for methylation sites and the clinical samples were relatively strict compared with the research by Hu et al. [38]. In addition to finding the CpGs which could predict the prognosis of patients with gastric cancer, we also made the gene functional enrichment analysis and found some key genes that were associated with patients’ clinical categories such as tumor stages.

Functional enrichment analysis of the corresponding genes for these methylation sites revealed that they were enriched in several pathways, with the highest enrichment in the pathway for pathways in cancer (Fig. 7a). Platelet-derived growth factor receptor alpha (PDGFRA) was shared in pathways of gastric cancer and hepatocellular carcinoma (Fig. 7b). To further select the key genes in the complex network, we analyzed all of genes integrating with the clinical categories and found that TGFβ2, GNG11, PDGFB, and ITGA5 were changed among different patients’ tumor stages, states, T categories, and grades (Fig. 7b, c). Previous studies showed that in lung squamous cell carcinoma GNG11 was a novel hub gene in module-related tumor size, and the low mRNA expression of GNG11 was associated with the higher overall survival rate for patients [39, 40]. The secretion of PDGFB by gastric carcinoma cells was associated with lymphatic metastasis [41]. For ITGA5, it was reported to be a potential diagnosis biomarker and therapeutic target for GC [42, 43]. The secretion of TGFβ2 by melanoma cells was essential for their metastases to the brain parenchyma [44]. In breast cancer, a novel functional polymorphism in TGFβ2 gene promoter could enhance TGFβ2 expression levels in vivo and therefore contribute to the tumor progression and the development of metastases [45]. For gliomas, Arslan et al. [46] showed that TGFβ2 could trigger the malignant phenotype of high-grade gliomas by inducing migration, and Zhang et al. [47] reported that there exited a potential mechanism of autophagy-associated glioma invasion that TGFβ2 could initiate autophagy via Smad and non-Smad pathway to promote glioma cells’ invasion. In gastric cancer, Yang et al. demonstrated that TGFβ2 played a vital role in linking epithelial–mesenchymal transition and tumor mutational burden, which suggested that TGFβ2 may be a predictive therapeutic target for GC [48]. Wang et al. [49] showed that the tumor-associated macrophages participated in GC cell invasion and metastasis through the TGFβ2/ nuclear factor kappa B /Kindlin-2 axis, providing a possibility for new treatment options and approaches. In this study, we found the level of TGFβ2 was not only significantly changed among different patients’ tumor stages, states, T categories, and grades as described above, but also up-regulated in patients with GC *vs* the normal (Additional file 12: Table S11, Additional file 13: Table S12, Fig. 7b, c). Moreover, it was involved in almost all of the 10 pathways as shown in Fig. 7b except p53 signaling pathway and melanoma, indicating that it might be an important factor which could affect the prognosis of patients with gastric cancer independently of p53 signaling pathway. Furthermore, among the 610 independent prognosis-related methylation sites, cg11976166 was located on the promoter region of TGFβ2, it might affect this gene’s expression and therefore influence its function. However, there were also limitations in this study. Although the established prognosis-predicting model had been validated in the testing group, there were few datasets which could be obtained to retest the efficiency of this model.

## Conclusion

We constructed a Cox risk regression model based on the independent prognosis-associated methylation sites, to distinguish patients in the high-risk (poor prognosis) and low-risk (relatively good prognosis) groups. This model was based on more than 400 DNA methylation samples of GC and was highly efficient in identifying high-risk patients. DNA methylation sites can be determined from noninvasive samples such as body fluids, which enables patients to avoid the pain associated with biopsy. In addition, the epigenetic levels in lesions were often altered before the expression of the genes, indicating that information about pathological changes in the tissues can be obtained earlier. Corresponding genes of these independent prognosis-associated methylation sites were mainly enriched in pathways in cancer. Among all of the genes TGFβ2 was emphasized and associated with patients’ prognosis or tumor progression, its expression could be affected by cg11976166. In the future, we plan to make the further insight into the mechanism of the critical genes and the key methylation sites in GC that were identified in this research.

## Supplementary information

**Additional file 1:** **Supplementary Table 1.** 406 patients' information and the follow-up data.

**Additional file 2:** **Supplementary Table 2.** Training matrix after data filtering and batch correction.

**Additional file 3:** **Supplementary Table 3.** Testing matrix after data filtering and batch correction..

**Additional file 4:** **Supplementary Table 4.** Prognosis-related methylation sites by univariate Cox regression analysis (1054 sites were found, *P* < 0.05).

**Additional file 5:** **Supplementary Table 5.** Multivariate Cox regression analysis of the 1054 methylation sites (610 sites were found, *P* < 0.05).

**Additional file 6:** **Supplementary Table 6.** Mehylation sites used in the Cox proportional hazard model and their coefficients.

**Additional file 7:** **Figure S1.** Comparison of clinical characteristics among different clusters. The comparison of the seven clusters in terms of clinical category T (**a**), N (**b**), and M (**c**), stage (**d**), grade (**e**), gender (**f**), and age (**g**). The unit for age is year. C, cluster; T, primary tumor; N, lymph node involvement; M, distant metastases; G, grade.

**Additional file 8:** **Supplementary Table 7.** Analysis of differences in methylation site levels between the 7 clusters (conMean, the mean methylation level in other clusters; treatMean, the mean methylation level in targeted cluster; FC, fold change).

**Additional file 9:** **Supplementary Table 8.** Risk assessment of training group.

**Additional file 10:** **Supplementary Table 9.** Risk assessment of testing group.

**Additional file 11:** **Supplementary Table 10.** Functional enrichment analysis of site genes.

**Additional file 12:** **Supplementary Table 11.** *P* values of the expression of site genes among different clinical categories

**Additional file 13:** **Supplementary Table 12.** Differently changed site genes between patients with gastric cancer and the healthy people

## Data Availability

The data that support the findings of this study are available from the corresponding author or the first author upon reasonable request.

## Abbreviations

GC: Gastric cancer
CpG: 5′-Cytosine-phosphate-guanine-3’
TGFβ2: Transforming growth factor β2
H. pylori: Helicobacter pylori
DNMT: DNA methyl-transferase
SAM: S-adenosyl-*L*-methionine
TCGA: The cancer genome atlas
UCSC: University of California Santa Cruz
T: Primary tumor
N: Lymph node involvement
M: Distant metastases
CDF: Cumulative distribution function
KEGG: The Kyoto Encyclopedia of Genes and Genomes
ANOVA: Analysis of variance
ROC: Receiver operating characteristic
ITGA5: Integrin subunit alpha 5
PDGFB: Platelet-derived growth factor subunit B
GNG11: G protein subunit gamma 11
NFE2L2: Nuclear factor, erythroid 2-like 2
GNA12: G protein subunit alpha 12
PDGFRA: Platelet-derived growth factor receptor alpha
C: Cluster
G: Grade
AUC: Area under the curve
CI: Confidence interval
*r*_*s*_: Coefficient of Spearman correlation analysis
