# Supplementary material for: Identification of the subtypes of gastric cancer based on DNA methylation and the prediction of prognosis
Source: Clin Epigenetics. 2020 Oct 28;12:161. doi: 10.1186/s13148-020-00940-3 (PMC7592597; doi:10.1186/s13148-020-00940-3)
Supplement: Supplementary file 7 — Additional file 7: Figure S1. Comparison of clinical characteristics among different clusters. The comparison of the seven clusters in terms of clinical category T (a), N (b), and M (c), stage (d), grade (e), gender (f), and age (g). The unit for age is year. C, cluster; T, primary tumor; N, lymph node involvement; M, distant metastases; G, grade. [file 13148_2020_940_MOESM7_ESM.docx]

**Supplemental Figures**


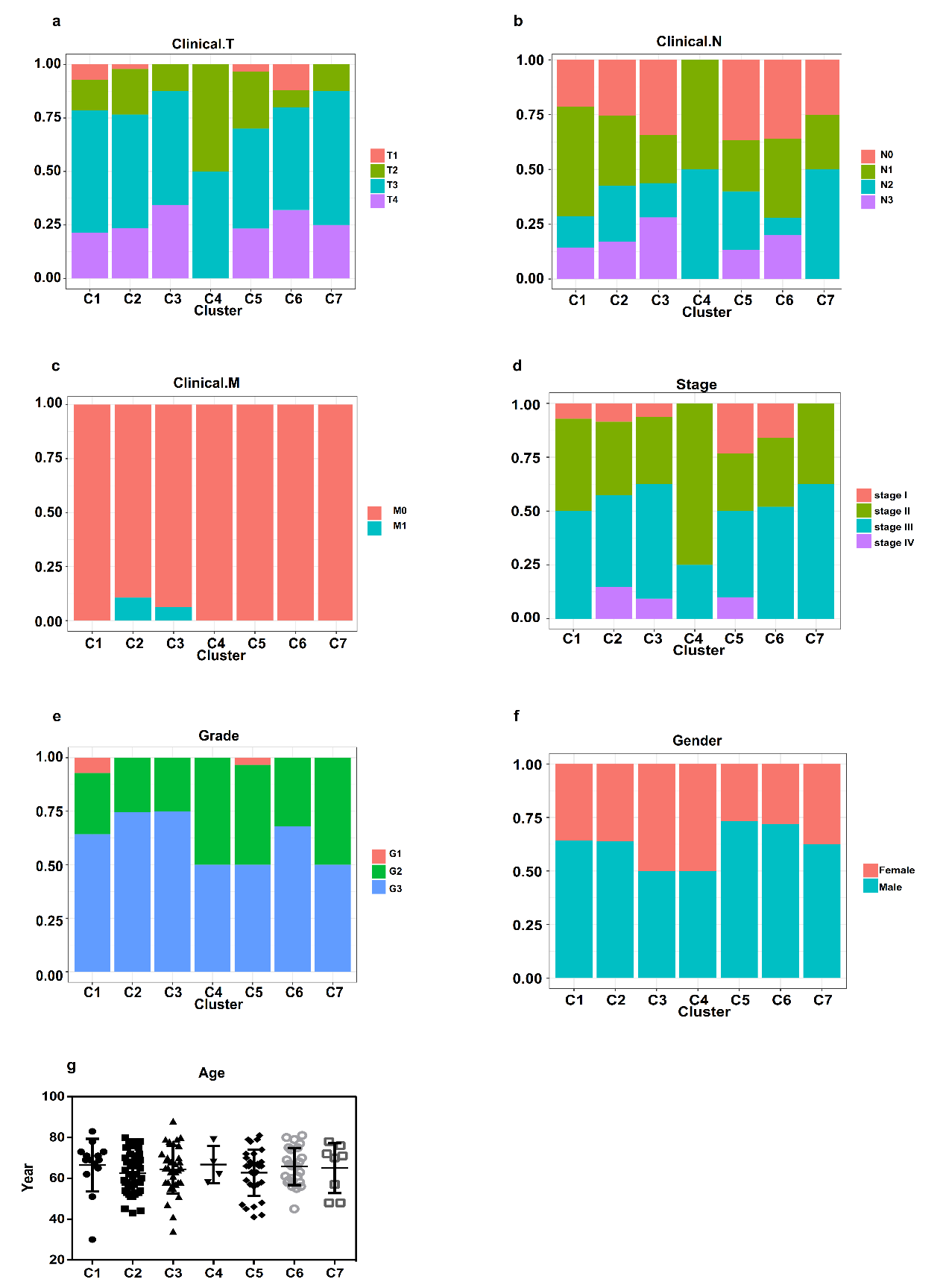


**Figure S1. Comparison of clinical characteristics among different clusters.** The comparison of the seven clusters in terms of clinical category T **(a)**, N **(b)**, and M **(c)**, stage **(d)**, grade **(e)**, gender **(f)** and age **(g)**. The unit for age is year. C, cluster; T, primary tumor; N, lymph node involvement; M, distant metastases; G, grade.
